# Atomic-Scale Engineering of Abrupt Interface for Direct Spin Contact of Ferromagnetic Semiconductor with Silicon

**DOI:** 10.1038/srep22841

**Published:** 2016-03-09

**Authors:** Dmitry V. Averyanov, Christina G. Karateeva, Igor A. Karateev, Andrey M. Tokmachev, Alexander L. Vasiliev, Sergey I. Zolotarev, Igor A. Likhachev, Vyacheslav G. Storchak

**Affiliations:** 1 National Research Center “Kurchatov Institute”, Kurchatov Square 1, Moscow 123182, Russia

## Abstract

Control and manipulation of the spin of conduction electrons in industrial semiconductors such as silicon are suggested as an operating principle for a new generation of spintronic devices. Coherent injection of spin-polarized carriers into Si is a key to this novel technology. It is contingent on our ability to engineer flawless interfaces of Si with a spin injector to prevent spin-flip scattering. The unique properties of the ferromagnetic semiconductor EuO make it a prospective spin injector into silicon. Recent advances in the epitaxial integration of EuO with Si bring the manufacturing of a direct spin contact within reach. Here we employ transmission electron microscopy to study the interface EuO/Si with atomic-scale resolution. We report techniques for interface control on a submonolayer scale through surface reconstruction. Thus we prevent formation of alien phases and imperfections detrimental to spin injection. This development opens a new avenue for semiconductor spintronics.

Impressive progress in data storage technology has arisen from the development of metallic spintronics based on giant magnetoresistance^1^. More recently, the focus has shifted towards spin transfer, semiconductor, molecular, and single-electron spintronics. In particular, semiconductor spintronics^2^ offers transistor action with the promise of hybrid logic, communications and storage devices as well as computing based on dissipationless spin transport. Driven initially by studies of GaAs, a material with strong spin-orbit coupling and efficient optical orientation of spins, it is now exploring the benefits of silicon^3^.

The cornerstones of semiconductor spintronics – creation, manipulation and detection of spin polarization – are extremely challenging in nonmagnetic Si. Optical orientation of spins is inefficient in silicon, motivating approaches based on electrical spin injection. Direct injection of spin-polarized electrons from a ferromagnetic metal into Si is ineffective due to the impedance mismatch^4^. Also, intermixing at the interface with formation of metal silicides hinders spin transport^5^. Moreover, interface roughness^6^ and magnetic domain structure^7^ reduce spin accumulation and enhance spin relaxation.

As metals are ineffective injectors, a number of alternatives have been proposed. The use of ferromagnetic tunnel contacts is thought to be a robust approach – the spin polarization of electrons thus injected into Si is detected using optical^8^, local^9^ and non-local^10^ electrical methods. The injection is highly sensitive to both the material of the barrier^11^ and its growth conditions^12^. Alternatively, spin currents can be induced by ballistic hot electrons^13^, dynamical^14^, thermal^15^, or acoustic^16^ injection. However, all these approaches need major advances to become technologically viable.

Spin injection technologies based on insulating tunnel barriers are plagued by high contact resistance at the interface: the next generation of spin MOSFETs would require a drastic reduction of the resistance between source and drain. The impedance mismatch problem suggests half-metallic^17^ or semiconductor injectors. A heterostructure of a ferromagnetic semiconductor in direct contact with a non-magnetic semiconductor would be a straightforward solution^18^. However, the problem of intermixing at the interface persists. Thus, a clean flawless interface between the semiconductors is indispensable.

EuO is a semiconductor with inherent ferromagnetism which can be tuned by strain^19^, doping^20^ or optical pumping^21^. Remarkable bulk properties – a metal-insulator transition accompanied by 13–15 orders of magnitude change in resistivity, colossal magnetoresistivity effect of 6 orders of magnitude in a magnetic field of 2 T, pronounced magneto-optics effects – distinguish EuO among magnetic materials. Being a magnetically homogeneous^22^ source of almost fully spin-polarized electrons^23^,^24^, it becomes a playground material for interfacial spin effects in theoretical simulations^25^,^26^,^27^. Among magnetic semiconductors, EuO is a leading candidate for integration with Si due to the materials’ structural and electronic compatibility and the thermodynamic stability of the EuO/Si contact^28^. However, despite tremendous efforts to integrate these semiconductors^29^,^30^,^31^,^32^,^33^,^34^,^35^,^36^, the epitaxial growth of EuO directly on silicon has not been accomplished until now. In a recent paper^37^ we advocated a significant revision of the growth procedure; this procedure has now been implemented. Magnetic and X-ray studies of the resulting films are highly encouraging, suggesting that the development of direct EuO/Si spin contacts may soon become practically feasible.

Here, we report a novel technology for integration of functional oxides with silicon which solves the long-standing problem of direct epitaxial growth of the EuO/Si structure. We demonstrate that interface engineering through surface reconstruction controls the outcome of the growth. High-resolution transmission electron microscopy (TEM) shows a clean atomically sharp EuO/Si interface without any alien phases, which were an insurmountable obstacle in previous attempts. Well-developed thickness fringes – coherent oscillations of X-ray diffraction (XRD) intensity – provide another strong confirmation of the atomically abrupt EuO/Si interface. Furthermore, TEM reveals the mechanism for relaxation of strains arising from the lattice mismatch between the semiconductors.

## Results and Discussion

Integration of ionic functional oxides with covalent Si is always challenging but an epitaxial growth of EuO directly on Si faces additional difficulties. Although both Si and EuO are cubic, the lattice mismatch is large (+5.6%). The most important problem, however, is the high chemical reactivity of the substances involved. First, an excess of oxygen leads to higher oxides Eu_3_O_4_ and Eu_2_O_3_. Both Eu and O_2_ react with the substrate forming europium silicide and silicon oxide, respectively, phases preventing direct contact between EuO and Si. High temperature may cause intermixing of EuO and Si at the interface. This is a most critical issue because alien phases at the interface are highly detrimental to spin injection. The same is true for integration of EuO with Si through a spacer preventing chemical reactions^31^ – a buffer layer between EuO and Si derails spin injection by reducing it to tunnelling. Thus, both protection of the Si surface from chemical processes at the interface and the growth regime of EuO directly on Si are of paramount importance.

The unreconstructed bare Si (001) surface constitutes a square array of atoms with two singly occupied dangling bonds. Then, pairs of Si atoms dimerize leaving one dangling bond per atom. Dimer rows along 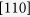 and 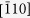 crystallographic directions correspond to a two-domain surface with 2 × 1 and 1 × 2 reconstruction domains (according to *in situ* reflection high-energy electron diffraction, RHEED). They stem from the two surface terminations of the Si lattice, separated by steps of an odd number of Si monolayers. Unpaired electrons make the surface highly susceptible to chemical reactions.

The Si surface with free valences saturated by metal atoms (typically Sr)^38^ is a standard template to grow functional oxides. Without surface states within the Si bandgap, the resulting 1 × 2 superstructure (submonolayer surface silicide SrSi_2_) is chemically resistant even if oxide growth transforms it into a surface silicate^39^,^40^. Such a Sr-passivated Si surface is a prerequisite for successful growth of alkaline earth and perovskite oxides^41^.

However, the 1 × 2 superstructure does not enable direct EuO/Si contact with subsequent epitaxial growth^35^. High resolution analytical scanning transmission electron microscopy (STEM) is an ultimate test of the quality of the interface. Recent atomic-resolution study of the EuO/Si interface with high-angle annular dark field (HAADF) scanning transmission electron microscopy accompanied by electron energy loss spectroscopy (EELS) reveals that EuO and Si are separated by several nm of harmful alien phases – either an unidentified disordered region or bulk europium silicide covered by and mixed with some Eu (III) non-magnetic phase(s)^35^ – a structure which precludes efficient spin injection. Similarly, silicide regions and a non-crystalline layer at the interface are detected by high-resolution TEM for EuO grown on H-passivated silicon^36^. Thus, both approaches suggested so far – Si surface passivation either by the 1 × 2 metal-based superstructure or by hydrogen – do not solve the problem of the formation of the direct EuO/Si contact. Moreover, H-passivation introduces further complications arising from an additional chemical element in the system. We find a deliberate engineering of the EuO/Si interface by a metal-based superstructure to be by far more advanced technique.

**Figure 1:**
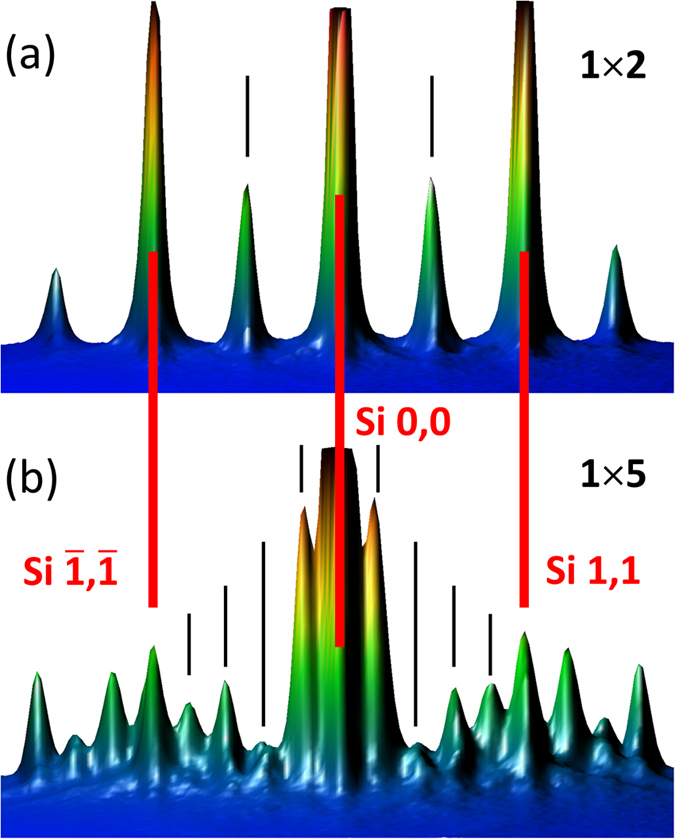
3D RHEED images for (**a**) 1 × 2 Eu/Si reconstruction and (**b**) 1 × 5 Eu/Si reconstruction. Red lines indicate positions of Si surface peaks while black lines show peaks coming from the reconstructions.

**Figure 2:**
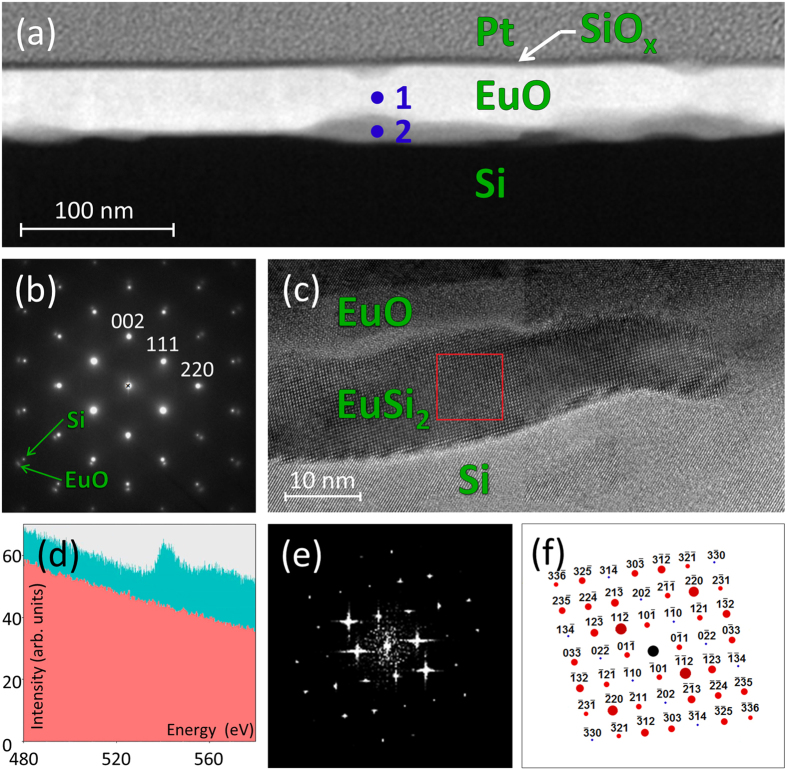
Structure of EuO/Si cross-section for 1 × 2 interface reconstruction. (**a**) Low-magnification cross-sectional HAADF-STEM image of EuO on Si protected by SiO_x_ and covered by Pt viewed along the [110] zone axis of both the EuO film and Si substrate, showing regions of stoichiometric EuO (point 1) and alien phases (point 2). (**b**) Selected area electron diffraction pattern (SADP) of EuO superimposed with that of Si revealing their relative orientation. (**c**) Cross-sectional HAADF-STEM image of the interface between EuO and Si demonstrating EuSi_2_ precipitate pocket. (**d**) Electron energy loss spectroscopy spectra for EuO and precipitate regions. Oxygen peaks detected for EuO (cyan area) are absent in the precipitates (red area). (**e**) Two-dimensional Fourier spectrum for the region marked on Fig. 1c by a red square. (**f**) SADP simulated for EuSi_2_ with the [111] zonal axis.

Following the metal-based superstructure paradigm we first tested the 1 × 2 reconstruction (Fig. 1a). To identify the problem we varied the growth conditions – the substrate temperature, the Eu beam and the oxygen pressure. It turns out that the intended EuO/Si contact is unattainable by these means. In particular, low temperature growth leads to disordered layers while higher temperature instigates chemical reactions at the interface. Figure 2 outlines a typical outcome of the growth. A cross-sectional HAADF-STEM image of the film at low magnification (Fig. 2a) shows the absence of the direct EuO/Si contact. Nevertheless, EuO is epitaxially integrated with Si. This fact is reflected on the selected area (electron) diffraction pattern (SADP) of EuO superimposed with that of Si (Fig. 2b) demonstrating the cube-on-cube orientation coupling. Energy dispersive X-ray spectroscopy (EDXS) certifies that the ratio Eu:O at point 1 is 1:1 within the method’s accuracy which proves EuO to be stoichiometric. In contrast, the by-product at point 2 is devoid of oxygen which signifies formation of a bulk silicide. The standardless quantitative EDXS analysis reveals the 1:2 stoichiometry of Eu:Si. A close-up look at the film (Fig. 2c) detects crystalline precipitates. Their linear size is up to 20 nm. In fact, the outcome of our attempts to achieve the direct EuO/Si contact based on the 1 × 2 reconstruction turns out to be unsuccessful to roughly the same extent as that of Mundy *et al.*^35^ – the result which clearly calls for an alternative approach.

The ternary phase diagram of the Eu-O-Si system is rich. Among binary compounds, oxides EuO, Eu_3_O_4_ and Eu_2_O_3_ (monoclinic and cubic), and silicides EuSi_2_ (tetragonal and hexagonal), Eu_3_Si_4_, EuSi, Eu_5_Si_3_ and Eu_2_Si may form. The known ternary compounds are Eu (II) orthosilicate Eu_2_SiO_4_ (monoclinic and orthorhombic) and Eu (III) disilicate Eu_2_Si_2_O_7_. Figure 2d shows EELS spectra for the EuO and precipitate regions: a peak characteristic for oxygen is well developed for EuO regions but is missing in the case of the precipitate. This fact indicates that the precipitate may be a binary Eu silicide. Figure 2e presents a two-dimensional Fourier spectrum from the region highlighted on Fig. 2c. Comparison with the known structures for bulk silicides assigns the image to tetragonal (I4_1_/amd, space group #141) EuSi_2_^42^ with the [111] zonal axis (the simulated SADP of EuSi_2_ matching the Fourier spectrum is given in Fig. 2f). It is worth to note that a detailed study of the film also reveals precipitate regions formed by the same tetragonal EuSi_2_ but oriented along the [201] axis.

The presence of precipitates calls for a better protection of the substrate surface. The formation of bulk Eu silicide suggests that the surface requires protection from Eu. On the other hand, surface reactions with oxygen which lead to disintegration of the substrate protection are equally important to suppress. As a consequence, we endeavoured to find a better way to prevent surface reactivity. The solution comes from our realization that metal superstructures other than 1 × 2 can be used for interfacing Si and oxide. The almost identical ionic radii of Eu^2+^ and Sr^2+^ ensure the well-known isomorphism of Eu (II) and Sr compounds with close structural parameters. One can expect similar phase diagrams for superstructures of these metals on the Si surface. Indeed, not only stoichiometric MSi_2_ but also equivalent 2 × 3 surface reconstructions are known for Eu and Sr^43^,^44^. The latter structure is not suitable for oxide epitaxy^43^ due to incomplete saturation of dangling bonds. It is more practical to use surface structures with a high metal coverage which have an advantage of full valence saturation and additional resistance with respect to oxidation. Such reconstructions (1 × 5 and 1 × 3) are known for Sr on Si (001).

The use of metal-rich silicide templates is in line with the original recipe for the growth of crystalline oxides on silicon^38^: the surface silicide is covered with an alkaline earth metal at low temperature to prevent oxidation of the silicide submonolayer at the initial stage of oxygen supply to the chamber. Instead, our intent is to provide an additional protection by chemically bound rather than physically adsorbed metal atoms. Following this route, we managed to get a stable, previously unknown 1 × 5 (according to RHEED, Fig. 1b) reconstruction of Eu on Si by subjecting the clean Si surface to a Eu flux at 660 °C. The spatial structure of surface silicides with the 1 × 5 reconstruction is not known; even the metal coverage of the surface is debated^45^, with estimates ranging from 0.8 to 1.5 monolayers. The only consensus reached so far is that the 1 × 5 reconstruction contains somewhat larger amount of metal than the well-known 1 × 2 SrSi_2_ structure. This fact suggests breaking of some surface Si dimers susceptible to oxidation and saturation of the broken bonds by metal atoms. It is likely that at the initial stage of the growth the 1 × 5 surface silicide is partially oxidized similar to that for the 1 × 2 reconstruction^40^.

**Figure 3:**
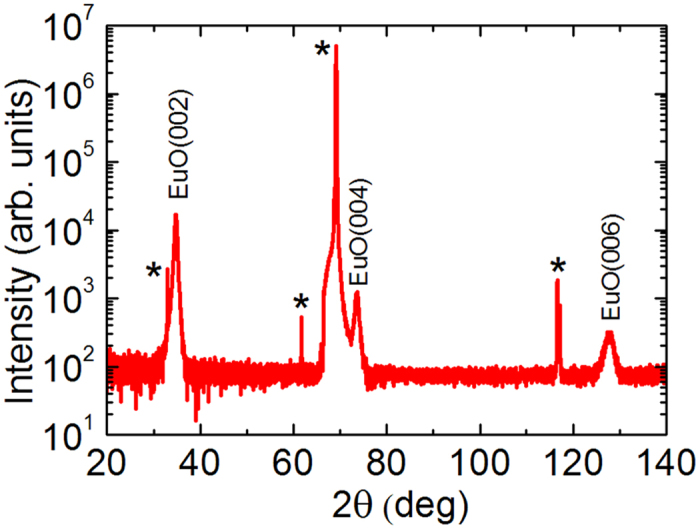
X-Ray diffraction θ–2θ spectrum of EuO/Si film for the 1 × 5 interface reconstruction. The image shows high-intensity EuO peaks (002), (004), and (006). Asterisks mark peaks from the Si substrate. No alien phases are detected.

**Figure 4:**
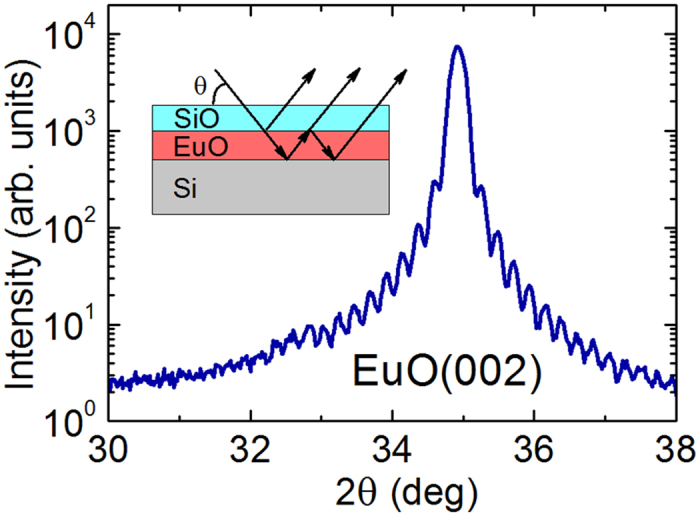
Thickness fringes around EuO (002) reflection in the θ–2θ XRD scan. Inset shows a model sketch of the wave interference resulting in the appearance of thickness fringes.

**Figure 5:**
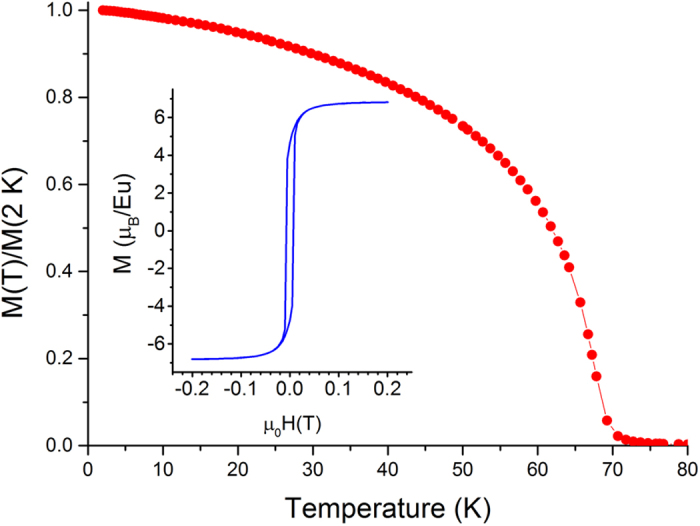
Temperature dependence of the normalized magnetization (measured at 100 Oe) of EuO/Si film grown on the 1 × 5 reconstruction showing *T*_*C*_ = 69 K. Inset: Magnetic field dependence of magnetization at *T* = 2 K. Hysteresis loop indicates the ferromagnetic state of EuO with the saturation magnetic moment 7 μ_B_ per Eu atom.

The replacement of the 1 × 2 by 1 × 5 reconstruction (Fig. 1) is a fundamental change of the growth technology dramatically affecting the outcome. Such a change may be beneficial even in the uncomplicated case of the growth of lattice-matched Ba_0.7_Sr_0.3_O on Si (001)^46^. As for complex cases like the EuO/Si contact, it turns out to be vital. Indeed, we witness a remarkable improvement of the quality of the EuO films. Characterization is carried out by a combination of different techniques. The x-ray diffraction θ–2θ spectrum (Fig. 3) shows well-developed peaks of EuO (200), (400) and (600) reflections without any signs of alien phases. This contrasts strongly with previous attempts^35^,^36^. In particular, integration of EuO with Si based on the 1 × 2 reconstruction results in films contaminated by the EuSi_2_ alien phase at the interface as revealed by XRD^35^. Moreover, well-resolved thickness fringes are observed not only for EuO (200) (Fig. 4) but also for EuO (400) and EuO (600) peaks^47^. This characteristic feature of x-ray diffraction is a result of wave interference due to reflections at the interfaces. As the value of the x-ray wave length (1.5418 Å) is smaller than the interatomic distance, the observation of thickness fringes is a fingerprint of atomically abrupt interfaces; otherwise the reflected waves cannot maintain the coherence and thickness fringes would not show up. This remarkable result not only points to the sharpness of the EuO interfaces but also reflects a superb structural quality of the film^48^. As far as we know, there are no reports by other groups of thickness fringes in the XRD pattern for EuO thin films. The respective positions of EuO and Si peaks on the (202) reflection φ-scan indicate that the vertical facets of EuO and Si are aligned in parallel. Magnetic measurements supported by Rutherford backscattering (RBS) estimation of Eu content in the films determine the moment of Eu atoms to be 7 μ_B_ while the Curie temperature (69 K) matches that of bulk EuO (Fig. 5). RBS itself reveals the perfect EuO stoichiometry. Furthermore, RBS spectra exhibit strong channeling – yet another indication of the epitaxial integration of EuO and Si. Transport measurements demonstrate highly insulating behaviour of the EuO films further confirming the perfect stoichiometry.

**Figure 6:**
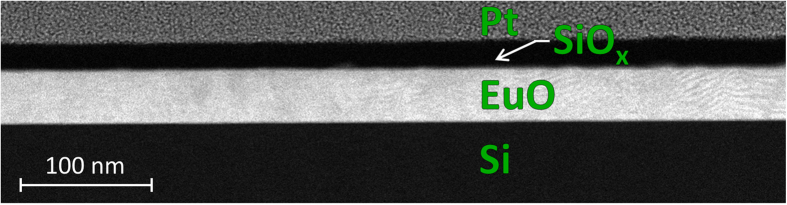
HAADF-STEM image of EuO/Si film for the 1 × 5 interface reconstruction. Low-magnification cross-sectional image viewed along the [110] zone axis of both the EuO film and Si substrate shows superb quality of EuO interfaces with both Si substrate and SiO_x_ capping.

**Figure 7:**
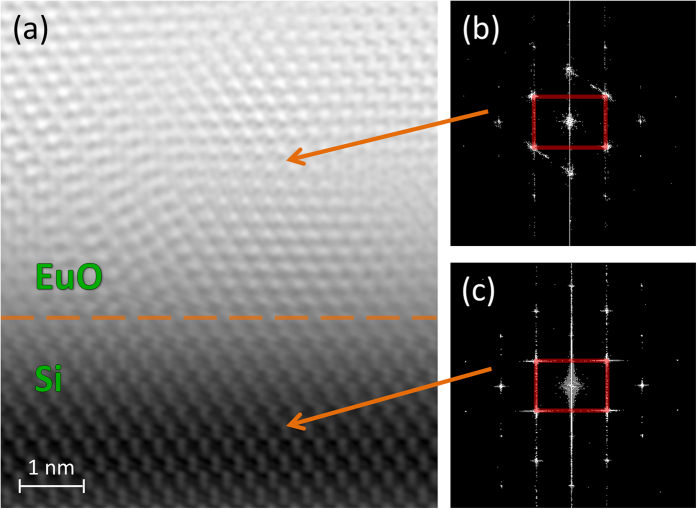
Structure of EuO/Si cross-section for the 1 × 5 interface reconstruction. (**a**) High-resolution cross-sectional HAADF-STEM image of EuO on Si viewed along the [110] zone axis of both the EuO film and Si substrate. Orange dashed line separates EuO and Si. (**b**) Two-dimensional Fourier spectrum for the EuO region of Fig. 7a. (**c**) Two-dimensional Fourier spectrum for the Si region of Fig. 7a.

**Figure 8:**
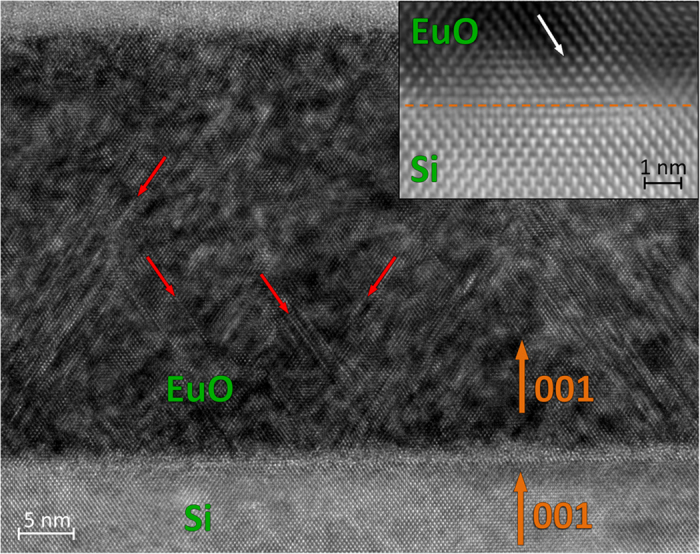
Relaxation of EuO film at the EuO/Si interface. Low-magnification bright-field cross-sectional image of EuO on Si viewed along the [110] zone axis of both the EuO film and Si substrate. Bold arrows show the 001 direction of both EuO and Si. Thin arrows point at several Shockley partial dislocations. Inset: high-resolution bright-field image for a fragment of the EuO/Si interface. Arrow indicates a Shockley partial dislocation.

The eye-catching effect of the technology change is revealed by electron microscopy. Figure 6 demonstrates a typical cross-sectional HAADF-STEM image at low magnification – no alien phases at the interface are detected (cf. Fig. 2a). A high-resolution HAADF-STEM image (Fig. 7) confirms that the crystalline EuO film is *in direct contact* with the Si (001) surface. Figure 7 demonstrates an atomically abrupt EuO/Si interface. This conclusion can also be drawn from a high-resolution bright-field TEM image of the same interface (inset of Fig. 8).

Thus, the long-standing problem of interfacing EuO with Si is solved. The atomically sharp interface unravels the reason for well-developed thickness fringes in XRD images^37^. It is quite possible that similar results can be obtained with the growth template formed by the 1 × 3 metal reconstruction – another metal-rich surface silicide. It remains to determine how the lattice mismatch between EuO and Si is relaxed. It is better seen on bright-field TEM images. Figure 8 shows a fragment of the EuO/Si interface with different magnification. Defects marked by arrows are identified as stacking faults associated with Shockley partial dislocations, typical to fcc structures. Stacking faults tend to terminate: crude estimates determine their density to halve over the thickness of the EuO film. This mechanism may explain relaxation of strains caused by the lattice mismatch between EuO and Si.

In summary, we have successfully demonstrated the epitaxial growth of the ferromagnetic semiconductor EuO directly on silicon. Electron microscopy reveals an atomically abrupt EuO/Si interface. This quantum leap in the quality of EuO films comes from interface engineering – the first monolayer is formed by the 1 × 5 metal superstructure instead of the standard 1 × 2 reconstruction. It strongly suppresses unwanted chemical reactions. However, the change of the reconstruction is not sufficient for engineering the direct EuO/Si contact. Diligent tuning of the growth conditions is indispensable.

Future research should address a number of questions: the usefulness of other reconstructions, benefits for the growth of other functional oxides on Si, the structure of the surface reconstructions, possible oxidation of the silicide during oxide growth, the effect of the silicide on the resulting electronic properties of the interface. We hope that this breakthrough in the material engineering will be followed by a successful spin injection through the manufactured spin contact.

## Methods

### Synthesis

EuO films are grown in Riber Compact 12 system for molecular beam epitaxy furnished with a UHV system comprising Gamma Vacuum Titan Ion Pump, cryopump Cryo-Torr 8 (Brooks CTI Cryogenics), a titanium sublimation pump and cryopanels cooled down by liquid nitrogen. The pressure of residual gases is less than 10^−10^ Torr. 4N Eu and SiO (for capping) are supplied from Knudsen cell effusion sources. Molecular oxygen (6N) flux is tuned with the gas flow system based on the mass flow controller and Baratron manometer. The cell and substrate temperatures are controlled with thermocouples while the absolute temperature of the substrate is determined with PhotriX ML-AAPX/090 infrared pyrometer (LumaSense Technologies) operating at the 0.9 μm wavelength. The intensity of molecular beams is measured with a Bayard-Alpert ionization gauge fitted at the substrate site.

The substrates are high-ohmic compensated Si (001) wafers with miscut angles not exceeding 0.5°. The natural surface oxide is removed by heating at 950 °C. The (2 × 1) reconstructed Si surface is exposed to a flux of Eu atoms at 660 °C until a stable (1 × 5) Eu/Si superstructure is formed. EuO films are grown at a temperature of 340 ± 10 °C. The oxygen pressure is about 6·10^−9^ Torr. The corresponding Eu flux comes from the effusion cell heated to 500 ± 10 °C. The growth rate at these conditions is determined to be ∼2 monolayers per minute. The surface of EuO is protected either by a capping layer of SiO or by controlled oxidation of its topmost layer with formation of Eu_2_O_3_. The quality of the films does not depend on the capping layer and does not deteriorate with time for at least one year.

### Transmission Electron Microscopy

The samples for analytical TEM/STEM are prepared in a Helios (FEI) scanning electron microscope (SEM)/Focus Ion Beam (FIB) dual beam system equipped with gas injectors for C and Pt deposition and a micromanipulator (Omniprobe). First, a 2 μm Pt layer is deposited on the surface of the sample. FIB milling (30 keV Ga^+^ ions) results in 2 μm thick cross-sections of approximately 8 × 5 μm^2^ area; then attached to the Omniprobe semiring. Electron transparency is achieved by further thinning and final cleaning with 5 keV and 2 keV Ga^+^ ion beams, respectively. The cross-sections are covered by thin C layers to prevent oxidation of EuO in the Helios chamber before breaking the vacuum. The specimens are studied with a 300 kV TEM/STEM Titan 80–300 (FEI), with a 0.8 Å STEM probe size and an EELS energy resolution of 0.75 eV. The microscope is equipped with a spherical aberration (Cs) corrector, a HAADF detector, an atmospheric thin-window energy dispersive X-ray spectrometer (Phoenix System, EDAX) and a post-column Gatan energy filter (GIF). Images are analysed with Digital Micrograph (Gatan) and Tecnai Imaging and Analysis (FEI) software.

### Characterization

The surface of the films is controlled *in situ* with reflection high-energy electron diffractometer fitted with kSA 400 Analytical RHEED system (k-Space Associates, Inc.). This system allows for a 3D representation of the RHEED pattern (Fig. 1) which displays the signal intensity as a third coordinate. Such representation is more informative than the standard 2D representation. X-ray diffraction experiments are carried out with Bruker D8 Advance and Rigaku SmartLab 9kW spectrometers (CuK_α_ X-ray source). Rutherford backscattering spectra are recorded for He ions with the energy 1.7 MeV. Magnetic properties are measured with SQUID magnetometer Quantum Design MPMS XL-7. Transport measurements are carried out using Lake Shore 9709A Hall effect measurement system.

## Additional Information

**How to cite this article**: Averyanov, D. V. *et al.* Atomic-Scale Engineering of Abrupt Interface for Direct Spin Contact of Ferromagnetic Semiconductor with Silicon. *Sci. Rep.* **6**, 22841; doi: 10.1038/srep22841 (2016).

## References

[b1] Fert A. Nobel lecture: Origin, development, and future of spintronics. Rev. Mod. Phys. 80, 1517–1530 (2008). 10.1002/anie.200801093 18626879

[b2] Awschalom D. D. & Flatté M. E. Challenges for semiconductor spintronics. Nature Phys. 3, 153–159 (2007).

[b3] Jansen R. Silicon spintronics. Nature Mater 11, 400–408 (2012). 22522640 10.1038/nmat3293

[b4] Schmidt G., Ferrand D., Molenkamp L. W., Filip A. T. & van Wees B. J. Fundamental obstacle for electrical spin injection from a ferromagnetic metal into a diffusive semiconductor. Phys. Rev. B 62, R4790–R4793 (2000).

[b5] Kioseoglou G. *et al.* Electrical spin injection into Si: A comparison between Fe/Si Schottky and Fe/Al_2_O_3_ tunnel contacts. Appl. Phys. Lett. 94, 122106 (2009).

[b6] Dash S. P. *et al.* Spin precession and inverted Hanle effect in a semiconductor near a finite-roughness ferromagnetic interface. Phys. Rev. B 84, 054410 (2011).

[b7] Ando Y. *et al.* Effect of the magnetic domain structure in the ferromagnetic contact on spin accumulation in silicon. Appl. Phys. Lett. 101, 232404 (2012).

[b8] Jonker B. T., Kioseoglou G., Hanbicki A. T., Li C. H. & Thompson P. E. Electrical spin-injection into silicon from a ferromagnetic metal/tunnel barrier contact. Nature Phys. 3, 542–546 (2007).

[b9] Dash S. P., Sharma S., Patel R. S., de Jong M. P. & Jansen R. Electrical creation of spin polarization in silicon at room temperature. Nature 462, 491–494 (2009). 19940922 10.1038/nature08570

[b10] van ‘t Erve O. M. J. *et al.* Electrical injection and detection of spin-polarized carriers in silicon in a lateral transport geometry. Appl. Phys. Lett. 91, 212109 (2007).

[b11] van ‘t Erve O. M. J. *et al.* Low-resistance spin injection into silicon using graphene tunnel barriers. Nature Nanotech. 7, 737–742 (2012). 10.1038/nnano.2012.161 23023645

[b12] Dankert A., Dulal R. S. & Dash S. P. Efficient spin injection into silicon and the role of the Schottky barrier. Sci. Rep. 3, 3196 (2013). 24217343 10.1038/srep03196 PMC3824168

[b13] Appelbaum I., Huang B. & Monsma D. J. Electronic measurement and control of spin transport in silicon. Nature 447, 295–298 (2007). 17507978 10.1038/nature05803

[b14] Shikoh E. *et al.* Spin-pump-induced spin transport in p-type Si at room temperature. Phys. Rev. Lett. 110, 127201 (2013). 25166836 10.1103/PhysRevLett.110.127201

[b15] Uchida K. *et al.* Observation of the spin Seebeck effect. Nature 455, 778–781 (2008). 18843364 10.1038/nature07321

[b16] Uchida K. *et al.* Long-range spin Seebeck effect and acoustic spin pumping. Nature Mater. 10, 737–741 (2011). 21857673 10.1038/nmat3099

[b17] Farshchi R. & Ramsteiner M. Spin injection from Heusler alloys into semiconductors: A materials perspective. J. Appl. Phys. 113, 191101 (2013).

[b18] Ohno Y. *et al.* Electrical spin injection in a ferromagnetic semiconductor heterostructure. Nature 402, 790–792 (1999).

[b19] Souza-Neto N. M., Haskel D., Tseng Y.-C. & Lapertot G. Pressure-induced electron mixing and enhancement of ferromagnetic ordering in EuX (X = Te, Se, S, O) magnetic semiconductors. Phys. Rev. Lett. 102, 057206 (2009). 19257546 10.1103/PhysRevLett.102.057206

[b20] Jutong N., Eckern U., Mairoser T. h. & Schwingenschlögl U. Effect of Gd doping and O deficiency on the Curie temperature of EuO. Sci. Rep. 5, 8038 (2015). 25623623 10.1038/srep08038 PMC4306962

[b21] Matsubara M. *et al.* Ultrafast optical tuning of ferromagnetism via the carrier density. Nature Commun. 6, 6724 (2015). 25832200 10.1038/ncomms7724

[b22] Storchak V. G. *et al.* Magnetic polarons in Eu-based films of magnetic semiconductors. Phys. Rev. B 81, 153201 (2010).

[b23] Steeneken P. G. *et al.* Exchange splitting and charge carrier spin polarization in EuO. Phys. Rev. Lett. 88, 047201 (2002). 11801161 10.1103/PhysRevLett.88.047201

[b24] Santos T. S. *et al.* Determining exchange splitting in a magnetic semiconductor by spin-filter tunnelling. Phys. Rev. Lett. 101, 147201 (2008). 18851564 10.1103/PhysRevLett.101.147201

[b25] Yang H. X. *et al.* Proximity effects induced by magnetic insulators: First-principles calculations on spin filtering and exchange splitting gaps. Phys. Rev. Lett. 110, 046603 (2013). 25166184 10.1103/PhysRevLett.110.046603

[b26] Zhang H., Wang J., Xu G., Xu Y. & Zhang S.-C. Topological states in ferromagnetic CdO/EuO superlattices and quantum wells. Phys. Rev. Lett. 112, 096804 (2014). 24655270 10.1103/PhysRevLett.112.096804

[b27] Garrity K. F. & Vanderbilt D. Chern insulator at a magnetic rocksalt interface. Phys. Rev. B 90, 121103(R) (2014).

[b28] Hubbard K. J. & Schlom D. G. Thermodynamic stability of binary oxides in contact with silicon. J. Mater. Res. 11, 2757–2776 (1996).

[b29] Lettieri J. *et al.* Epitaxial growth and magnetic properties of EuO on (001) Si by molecular-beam epitaxy. Appl. Phys. Lett. 83, 975–977 (2003).

[b30] Santos T. S. & Moodera J. S. Observation of spin filtering with a ferromagnetic EuO tunnel barrier. Phys. Rev. B 69, 241203(R) (2004).

[b31] Schmehl A. *et al.* Epitaxial integration of the highly spin-polarized ferromagnetic semiconductor EuO with silicon and GaN. Nature Mater. 6, 882–887 (2007). 17873862 10.1038/nmat2012

[b32] Beukers J. N. *et al.* Epitaxial EuO thin films by pulsed laser deposition monitored by *in situ* X-ray photoelectron spectroscopy. Thin Solid Films 518, 5173–5176 (2010).

[b33] Caspers C. *et al.* Chemical stability of the magnetic oxide EuO directly on silicon observed by hard X-ray photoemission spectroscopy. Phys. Rev. B 84, 205217 (2011).

[b34] Caspers C. *et al.* Ultrathin magnetic oxide EuO films on Si(001) using SiO_x_ passivation – controlled by hard X-ray photoemission spectroscopy. J. Appl. Phys. 113, 17C505 (2013).

[b35] Mundy J. A. *et al.* Hetero-epitaxial EuO interfaces studied by analytic electron microscopy. Appl. Phys. Lett. 104, 091601 (2014).

[b36] Caspers C. *et al.* Controlling the EuO interface – epitaxial EuO spin contacts directly on Si. arXiv: 1504.05108v1 2015.

[b37] Averyanov D. V. *et al.* Direct epitaxial integration of the ferromagnetic semiconductor EuO with silicon for spintronic applications. ACS Appl. Mater. Interf. 7, 6146–6152 (2015). 10.1021/am5089007 25723051

[b38] McKee R. A., Walker F. J. & Chisholm M. F. Crystalline oxides on silicon: The first five monolayers. Phys. Rev. Lett. 81, 3014–3017 (1998).

[b39] Först C. J., Ashman C. R., Schwarz K. & Blöchl P. E. The interface between silicon and a high-*k* oxide. Nature 427, 53–56 (2004). 14702081 10.1038/nature02204

[b40] Segal Y. *et al.* Atomic structure of the epitaxial BaO/Si(001) interface. Phys. Rev. Lett. 102, 116101 (2009). 19392218 10.1103/PhysRevLett.102.116101

[b41] Reiner J. W. *et al.* Crystalline oxides on silicon. Adv. Mater. 22, 2919–2938 (2010). 20432223 10.1002/adma.200904306

[b42] Evers J., Oehlinger G. & Weiss A. Effect of pressure on the structures of divalent metal disilicides MSi_2_ (M = Ca, Eu, Sr). J. Solid State Chem. 20, 173–181 (1977).

[b43] Reiner J. W., Garrity K. F., Walker F. J., Ismail-Beigi S. & Ahn C. H. Role of strontium in oxide epitaxy on silicon (001). Phys. Rev. Lett. 101, 105503 (2008). 18851225 10.1103/PhysRevLett.101.105503

[b44] Kuzmin M., Perälä R. E., Laukkanen P. & Väyrynen I. J. Atomic geometry and electronic structure of the Si(100)2 × 3-Eu surface phase. Phys. Rev. B 72, 085343 (2005).

[b45] Goodner D. M. Atomic-scale surface studies of alkaline-earth metals on Si(001). PhD thesis, Northwestern University (2005).

[b46] Zachariae J. & Pfnür H. Growth conditions, stoichiometry, and electronic structure of lattice-matched SrO/BaO mixtures on Si(100). Phys. Rev. B 72, 075410 (2005).

[b47] Averyanov D. V. *et al.* Structural coupling across the direct EuO/Si interface. Nanotechnology 27, 045703 (2016). 26655284 10.1088/0957-4484/27/4/045703

[b48] Bowen D. K. & Tanner B. K. High-Resolution X-Ray Diffractometry and Topography (Taylor & Francis, London, 2005, 278 p).

